# Periodontitis Is Associated With Heart Failure: A Population-Based Study (NHANES III)

**DOI:** 10.3389/fphys.2022.854606

**Published:** 2022-04-20

**Authors:** Yan Yan, Min Mao, Yan-Qin Li, Yong-Ji Chen, He-Dong Yu, Wen-Zhong Xie, Qiao Huang, Wei-Dong Leng, Jie Xiong

**Affiliations:** ^1^ Department of Stomatology, Taihe Hospital, Hubei University of Medicine, Shiyan, China; ^2^ Department of Stomatology, Kaifeng University Health Science Center, Kaifeng, China; ^3^ Center for Evidence-Based and Translational Medicine, Zhongnan Hospital of Wuhan University, Wuhan, China

**Keywords:** periodontitis, periodontal diseases, heart failure, cardiovascular diseases, NHANES

## Abstract

**Objectives:** The aim of this study was to investigate the relationship between periodontitis and heart failure using the Third National Health and Nutrition Examination Survey (NHANES III).

**Methods:** Participants who had received a periodontal examination were included and investigated for the occurrence of heart failure. The included participants were divided into no/mild periodontitis and moderate/severe periodontitis groups according to their periodontal status. Weighted prevalence of heart failure was calculated, and weighted logistic regressions models were used to explore the association between periodontitis and heart failure. Possible influencing factors were then explored through subgroup analysis.

**Results:** Compared with that of the no/mild periodontitis group, the incidence of heart failure in participants with moderate/severe periodontitis was 5.72 times higher (95% CI: 3.76–8.72, *p* < 0.001). After adjusting for gender, age, race, body mass index, poverty income ratio, education, marital status, smoking status, drinking status, hypertension, diabetes, stroke, and asthma, the results showed that the incidence of heart failure in the moderate/severe group was 3.03 times higher (95% CI: 1.29–7.13, *p* = 0.012). Subgroup analysis showed that criteria, namely, male, 40–60 years old, non-Hispanic white, body mass index >30, poverty income ratio ≥1, not more than 12 years of education, currently drinking, stroke but no diabetes, or asthma supported moderate/severe periodontitis as a risk factor for heart failure (*p* < 0.05).

**Conclusion:** According to data from this nationally representative sample from the United States, periodontitis is associated with an increased risk of heart failure.

## Introduction

Heart failure is a severe manifestation or late stage of various heart diseases. It is estimated that 64.3 million people worldwide suffer from heart failure (GBD 2017 Disease and Injury Incidence and Prevalence Collaborators, 2018). In developed countries, the prevalence of heart failure is generally 1–2% (Christiansen et al., 2017; Conrad et al., 2018; Smeets et al., 2019; Groenewegen et al., 2020). Heart failure can lead to cardiac arrest resulting in death unless there is timely and successful performance of CPR and it causes a large disease burden. The number of hospitalizations associated with heart failure has tripled in the past 30 years (Braunwald, 2013; Arrigo et al., 2020; Gu et al., 2020). According to the latest survey by the Heart Failure Association of the European Society of Cardiology, the median heart failure prevalence was 17.20 cases per 1,000 people (Seferović et al., 2021). A study using the National Inpatient Sample in the United States found that the hospitalizations due to heart failure increased from 1,060,540 in 2008 to 1,270,360 in 2018 (Clark et al., 2022). In addition to cardiovascular diseases such as ischemic heart disease, hypertension, and arrhythmia, inflammation also plays an important role in heart failure (Pieske et al., 2019; Groenewegen et al., 2020; Schiattarella et al., 2020). Studies have shown that inflammatory factors can lead to ventricular remodeling, which in turn affects the development of heart failure (Al-Taweel et al., 2017; Tan et al., 2021; Yan et al., 2021). In addition, there are also animal studies and clinical studies focusing on the positive effects of anti-inflammatory treatments on cardiovascular diseases (Bäck and Hansson, 2015; Huang and Frangogiannis, 2018; Ma et al., 2019; Gao et al., 2021).

Periodontitis is described as a chronic inflammation of the periodontal support tissue caused by local factors with severe periodontitis bringing a serious burden of disease (Sanz et al., 2020a; Luo et al., 2021). Evidence points to increased levels of systemic inflammatory mediators in periodontitis patients, such as C-reactive protein, matrix metalloproteinases, and fibrinogen (Schenkein et al., 2020). Previous studies have also reported the molecular mechanisms of inflammatory factors in the occurrence of cardiovascular diseases such as hypertension, atherosclerosis, and myocardial infarction (Prabhu and Frangogiannis, 2016; Guzik and Touyz, 2017; Moriya, 2019; Sardu et al., 2020). In addition, there are evidences to support a causal relationship between severe periodontitis and cardiovascular disease (Leng et al., 2015; Zeng et al., 2016; Czesnikiewicz-Guzik et al., 2019; Zhao et al., 2019; Sanz et al., 2020b). Heart failure is the terminal stage of cardiovascular disease, and few studies link periodontitis to it. Although a small number of studies have reported the relationship between periodontitis and heart failure (Wood and Johnson, 2004; Chen et al., 2016; Fröhlich et al., 2016), no in-depth statistical analysis of the association has been performed. Hence, we retrieved data from the Third National Health and Nutrition Examination Survey (NHANES III) to provide evidence demonstrating the relationship between periodontitis and heart failure.

## Methods

### Data and Participants

All data were from NHANES III, a nationwide survey which was conducted in America, in 1988–1994 and included 39695 persons aged 2 months and older (Ezzati et al., 1992; National Center for Health Statistics, 1994). The inclusion criteria for this study were people older than 18 years with clinical dental examination information. Individuals who did not have complete information about periodontal assessments and heart failure events were excluded. The relevant information of all participants was collected by well-trained examiners, including gender, age, race, body mass index (BMI, kg/m^2^), poverty income ratio, educational status, marital status, personal history, and related medical history. Then, participants were divided into no/mild periodontitis and moderate/severe periodontitis groups according to their periodontal status. The NHANES III was approved by the Institutional Review Board (IRB), and documented consent was obtained from participants (National Center for Health Statistics, 2017). This study used public data in NHANES III for analysis.

### Outcome

The occurrence of heart failure incidents was self-reported as “Yes” for the question about heart failure in the questionnaire (“Has a doctor ever told you that you had congestive heart failure?”) (Parikh et al., 2011; Sattler et al., 2019).

### Definitions of Periodontitis

As in previous studies, periodontal status was assessed by using the extent and severity of probing depth (PD) and attachment loss (AL) (Albandar et al., 1999; Page and Eke, 2007): no periodontitis: no evidence of mild, moderate, or severe periodontitis; mild periodontitis: ≥ two interproximal sites with attachment loss (AL) ≥3 mm and <4 mm and ≥ two interproximal sites with PD ≥ 4 mm not on the same tooth or one site with PD ≥ 5 mm; moderate periodontitis: ≥ two interproximal sites with AL ≥ 4 mm and <6 mm not on the same tooth or ≥ two interproximal sites with PD ≥ 5 mm not on the same tooth; severe periodontitis: ≥ two interproximal sites with AL ≥ 6 mm not on the same tooth and ≥one or more interproximal sites with PD ≥ 5 mm.

### Covariates

Information about gender, age, race, education, marital status, smoking history, drinking history, hypertension, diabetes, stroke, and asthma was collected through standardized household questionnaires. Race was classified as non-Hispanic white, non-Hispanic black, Mexican–American, and others. The education level was divided into ≤ 12 years and > 12 years. The marital status was divided into married, widowed/divorced/separated, and never married. Smoking was classified as never-smokers, former smokers, and current smokers. Drinking was classified as never drinking and currently drinking. Blood pressure was classified as high/uncontrolled blood pressure and normal/controlled blood pressure. The diabetes status was classified as diabetes, pre-diabetes, and non-diabetic. Stroke and asthma were classified as yes or no. BMI was calculated based on a person’s weight and height. The poverty income ratio is the ratio of a family’s income to the United States Census Bureau’s poverty threshold, which varies with the number and ages of family members and is revised yearly.

### Statistical Analysis

The participants were classified into two groups (no/mild periodontitis group and moderate/severe group). Basic characteristics were described and compared. Categorical variables were represented by counts and percentages and were compared using the weighted chi-square test, including gender, race, education, marital status, smoking history, drinking history, presence/absence of hypertension, diabetes, stroke, and asthma. Continuous variables were represented by mean ± standard deviation and compared using the weighted one-way ANOVA test, including age and BMI.

Weighted prevalence of heart failure and corresponding 95% confidence interval were estimated for the total participants and each group, respectively. To explore the potential and independent association between degrees of periodontitis and heart failure, a series of weighted logistic regressions were performed. Model 1 is an unadjusted model; Model 2 is adjusted for gender, age, race, BMI, and poverty income ratio; Model 3 is further adjusted for education, marital status, smoking status, and drinking status based on Model 2; Model 4 is further adjusted for hypertension, diabetes, stroke, and asthma based on Model 3. Collinearity was checked for each variable using the variance inflation factor (VIF), and VIF <10 was acceptable. In each model, the degree of periodontitis was considered a nominal variable, and the no/mild periodontitis group was used as the reference. To explore potential heterogeneity in the association, a series of subgroup analyses based on included characteristics were conducted.

The NHANES data adopted a “complex sample” strategy to collect data, which included study design weights, clusters weights, and stratification weights. All statistical analyses were survey weighted and were performed using SAS software, version 9.4 TS1M6 (SAS Institute Inc., Cary, NC). A two-sided *p*-value less than 0.05 is considered statistically significant.

## Results

### General Characteristics

In this study, a total of 33603 participants were downloaded from the NHANES database. A total of 404 participants who had missing values in strata and cluster variables were excluded. Of the remaining, 15811 participants were excluded because they were older than 18 or weight ≤0; 4,186 participants were excluded because of missing data in periodontitis or heart failure. Finally, 13202 participants were included in descriptive analysis. Characteristics of the 13202 participants are presented in Table 1. The participants are 43.02 ± 17.72 years old, including 6,234 males and 6,968 females, with a BMI of 27.10 ± 5.85. According to the periodontal condition of the participants, they were divided into no/mild periodontitis (*n* = 10606) and moderate/severe periodontitis groups (*n* = 2,596). Except for drinking (*p* = 0.139) and asthma (*p* = 0.894), all factors including gender, age, race, BMI, poverty income ratio, years of education, marital status, smoking, hypertension, diabetes, and stroke were significantly different among the two groups (*p* < 0.001).

**TABLE 1:** Weighted comparison in basic characteristics.

	Total	No/mild	Moderate/severe	*P* ^a^
	(*n* = 13202)	(*n* = 10606)	(*n* = 2,596)	
Sex				<0.001
Male	6,234 (47.22%)	4,653 (43.87%)	1,581 (60.90%)	
Female	6,968 (52.78%)	5,953 (56.13%)	1,015 (39.10%)	
Age (years)	43.02 ± 17.72	39.62 ± 16.40	56.90 ± 16.07	<0.001
Race				<0.001
Non-Hispanic white	4,826 (36.56%)	3,865 (36.44%)	961 (37.02%)	
Non-Hispanic black	3,814 (28.89%)	2,994 (28.23%)	820 (31.59%)	
Mexican–American	4,005 (30.34%)	3,299 (31.11%)	706 (27.20%)	
Others	557 (4.22%)	448 (4.22%)	109 (4.20%)	
BMI	27.10 ± 5.85	27.00 ± 5.87	27.50 ± 5.74	<0.001
PIR	2.44 ± 1.79	2.47 ± 1.79	2.31 ± 1.76	0.015
Education (years)				<0.001
≤12	9,032 (68.88%)	6,988 (66.32%)	2044 (79.38%)	
>12	4,080 (31.12%)	3,549 (33.68%)	531 (20.62%)	
Marriage				<0.001
Married	8,115 (61.58%)	6,465 (61.07%)	1,650 (63.68%)	
Widowed/divorced/separated	2,344 (17.79%)	1,637 (15.46%)	707 (27.29%)	
Never married	2,719 (20.63%)	2,485 (23.47%)	234 (9.03%)	
Smoking				<0.001
Never	6,838 (51.80%)	5,900 (55.63%)	938 (36.13%)	
Previous	2,915 (22.08%)	2,127 (20.06%)	788 (30.35%)	
Current	3,448 (26.12%)	2,578 (24.31%)	870 (33.51%)	
Drinking				0.139
Never	2,228 (26.50%)	1811 (26.18%)	417 (27.99%)	
Current	6,179 (73.50%)	5,106 (73.82%)	1,073 (72.01%)	
Hypertension				<0.001
High/uncontrolled BP	6,264 (47.45%)	4,566 (43.05%)	1,698 (65.41%)	
Normal/controlled BP	6,938 (52.55%)	6,040 (56.95%)	898 (34.59%)	
Diabetes				<0.001
Diabetes	1,182 (9.29%)	746 (7.29%)	436 (17.43%)	
Pre-diabetes	2,265 (17.79%)	1,552 (15.18%)	713 (28.50%)	
No	9,282 (72.92%)	7,929 (77.53%)	1,353 (54.08%)	
Stroke				<0.001
Yes	225 (1.70%)	129 (1.22%)	96 (3.70%)	
No	12972 (98.30%)	10474 (98.78%)	2,498 (96.30%)	
Asthma				0.894
Yes	896 (6.79%)	731 (6.89%)	165 (6.36%)	
No	12306 (93.21%)	9,875 (93.11%)	2,431 (93.64%)	

BMI, body mass index; PIR, poverty income ratio.

a Weighted comparison.

### Prevalence of Heart Failure

The prevalence of heart failure was tested. The results showed that among the 13202 participants, a total of 263 heart failure events were reported. There were significant differences in the prevalence of heart failure among no/mild periodontitis and moderate/severe periodontitis groups (*p* < 0.001), which were 0.64 and 3.55%, respectively. The weighted prevalence of heart failure is summarized in Table 2.

**TABLE 2:** Weighted prevalence of heart failure.

Group	Event/total	Weighted event/total	Prevalence (%)	95% CI^a^	*P*
Total	263/13202	1529319/143086481	1.07	0.83, 1.31	
No/mild periodontitis	153/10606	779581/121970978	0.64	0.45, 0.83	<0.001
Moderate/severe periodontitis	110/2,596	749738/21115503	3.55	2.38, 4.72	

a Based on weighted event/total.

### Associations Between Periodontitis and Heart Failure

We then analyzed the potential impact of periodontitis on the occurrence of heart failure by adjusting for different confounding factors. For the unadjusted model, the maximum value of VIF was 1.789, which indicated weak collinearity; compared with the no/mild periodontitis group, participants in the moderate/severe group had a 5.72 times higher incidence of heart failure (95% CI: 3.76–8.72, *p* < 0.001). After adjusting for gender, age, race, BMI and poverty income ratio, the incidence of heart failure in the moderate/severe group is still significantly higher than that in the no/mild periodontitis group (OR = 1.77, 95% CI: 1.02–3.06, *p* = 0.043). After further adjusting for education, marital status, smoking status, and drinking status, the incidence of heart failure in the moderate/severe group was still higher than that in the no/mild periodontitis group (OR = 2.45, 95% CI: 1.17–4.95, *p* = 0.018). After further correction for hypertension, diabetes, stroke, and asthma, participants in the moderate/severe group had a 3.03 times higher incidence of heart failure (95% CI: 1.29–7.13, *p* = 0.012). The summary results are shown in Table 3.

**TABLE 3:** Weighted logistic regressions of associations between periodontal status and heart failure.

Model	No/mild	Moderate/severe		
		OR	95% CI	*P*
Model 1	Reference	5.72	3.76, 8.72	<0.001
Model 2	Reference	1.77	1.02, 3.06	0.043
Model 3	Reference	2.45	1.17, 4.95	0.018
Model 4	Reference	3.03	1.29, 7.13	0.012

Model 1: unadjusted.

Model 2: adjusted for gender, age, race, body mass index, and poverty income ratio.

Model 3: further adjusted for education, marital status, smoking status, and drinking status based on Model 2.

Model 4: further adjusted for hypertension, diabetes, stroke, and asthma based on Model 3.

### Subgroup Analysis

We further explored the correlation between periodontal status and heart failure among different subgroups based on gender, age, race, BMI, poverty income ratio, education, marital status, smoking, drinking, hypertension, diabetes, stroke, and asthma. The results showed that participants who are males, 40–60 years old, non-Hispanic whites, BMI >30, poverty income ratio ≥1, no more than 12 years of education, current drinking, stroke but no diabetes, or asthma in the moderate/severe group are at risk of developing heart failure (*p* < 0.05). Regarding whether the situation is married or other, high/uncontrolled blood pressure or non-hypertension, the incidence of heart failure in the moderate/severe group is higher than that in the no/mild periodontitis group (*p* < 0.05). The results of subgroup analysis are shown in Table 4.

**TABLE 4:** Subgroup analysis.

Subgroup	No/mild	Moderate/severe		
		OR	95% CI	*P*
Sex				
Male	Reference	3.83	1.21, 11.97	0.02
Female	Reference	1.33	0.55, 3.40	0.54
Age				
<40	Reference	0.44	0.05, 4.39	0.48
40–60	Reference	7.04	2.47, 20.10	< 0.001
>60	Reference	2.14	0.85, 5.36	0.10
Race				
Non-Hispanic white	Reference	3.67	1.14, 11.78	0.03
Non-Hispanic black	Reference	1.33	0.54, 3.29	0.53
Mexican–American	Reference	1.22	0.55, 2.77	0.61
BMI				
<25	Reference	5.31	0.62, 45.93	0.13
25–30	Reference	2.32	0.93, 5.79	0.07
>30	Reference	3.49	1.04, 11.70	0.04
PIR				
<1	Reference	2.15	0.93, 4.94	0.07
≥1	Reference	3.30	1.02, 10.63	0.04
Edu				
≤12	Reference	2.69	1.25, 5.75	0.01
>12	Reference	3.53	0.35, 31.90	0.29
Marital status				
Married	Reference	2.93	1.05, 8.18	0.04
Other	Reference	3.08	1.37, 6.97	0.007
Smoking				
Never	Reference	4.49	0.89, 22.74	0.07
Previous/current	Reference	2.28	0.89, 5.85	0.08
Drinking				
Never	Reference	1.42	0.59, 3.41	0.42
Current	Reference	3.60	1.27, 10.20	0.02
Hypertension				
High/uncontrolled BP	Reference	2.14	1.05, 4.38	0.04
No	Reference	20.35	4.80, 85.88	<0.001
Diabetes				
Diabetes/pre-diabetes	Reference	1.56	0.72, 3.94	0.28
No	Reference	5.04	1.88, 13.48	0.001
Stroke				
Yes	Reference	34.42	2.44, 485.99	0.01
No	Reference	1.94	0.98, 3.85	0.06
Asthma				
Yes	Reference	0.39	0.07, 2.26	0.28
No	Reference	3.35	1.35, 8.34	0.01

BMI, body mass index; PIR, poverty income ratio; Edu, education.

## Discussion

Based on 10606 participants with no/mild periodontitis and 2,596 participants with moderate/severe periodontitis, this study investigated the risk of heart failure and its influencing factors in periodontitis patients. Our findings indicate that moderate/severe periodontitis shows a trend associated with an increased risk of heart failure, especially in which participants are males, 40–60 years old, non-Hispanic whites, BMI>30, poverty income ratio ≥1, no more than 12 years of education, drinking, stroke but no diabetes, or asthma. Moderate/severe periodontitis was a risk factor for heart failure regardless of marital status and hypertension. After adjusting for potential confounding factors, there is still an association trend between heart failure and moderate/severe periodontitis.

This study found that the weighted prevalence of heart failure in the moderate/severe periodontitis group is significantly higher than that in the no/mild periodontitis group. In total, three previous studies have mentioned the relationship between periodontitis and heart failure. Wood and Johnson (2004) had first reported the association between these two diseases. They evaluated more than 17,000 people from NHANES III and found that patients with periodontitis have a higher incidence of self-reported heart failure. The results are similar to our research, but no further analysis has been performed. Fröhlich et al. (2016) conducted a dental evaluation of 71 patients with stable chronic heart failure and found that the incidence of severe periodontitis was higher in patients with heart failure than those in the control group. A large Asian study using the Taiwan National Health Insurance Research Database reported a positive correlation between periodontitis and heart failure (Chen et al., 2016).

Heart failure is a clinical syndrome of functional failure having multiple causes, with high all-cause in-hospital or ICU mortality (van der Meer et al., 2019; Yu et al., 2021). Although there are differences in etiology and pathophysiology, it is characterized by chronic and unregulated inflammatory conditions, leading to progression of myocardial damage, decreased function, and poor prognosis (Dick and Epelman, 2016; Adamo et al., 2020). The global cardiovascular mortality report pointed out that many chronic infectious and inflammatory diseases, such as, interestingly, periodontitis, are associated with a significantly increased risk of cardiovascular adverse events (such as atrial fibrillation, myocardial infarction, and heart failure), (Roth et al., 2015). Periodontitis shows a high expression of inflammatory mediators and markers, implying a high level of systemic inflammatory factors (Pink et al., 2015; Sung et al., 2019; Fang et al., 2021a; Yuan et al., 2021). A study involving 13,784 subjects, based on NHANES III, has shown that severe periodontitis is independently and significantly related to cardiovascular mortality in chronic kidney disease (Sharma et al., 2016). Periodontitis and heart failure have many common risk factors, such as age, gender, smoking, diabetes, hypertension, and hypercholesterolemia (Mucci et al., 2009; Carrizales-Sepúlveda et al., 2018). After adjusting for various confounding factors, such as age, gender, and race, this study found that there is still an association between periodontitis and the risk of heart failure.

Furthermore, subgroup analysis in this study showed that moderate/severe periodontitis is a risk factor for heart failure, regardless of the presence or absence of hypertension, and the risk is higher in the non-hypertensive subgroup. This further shows that periodontitis may be a direct influencing factor of heart failure; in other words, periodontitis is a very important non-cardiovascular factor in the occurrence of heart failure. The mechanisms related to periodontitis and cardiovascular disease may be complicated (Sanz et al., 2020b). In general, the possible mechanisms involve the production and/or increased levels of inflammatory mediators caused by bacteremia, abnormal blood lipid metabolism, bacterial products and virulence factors, and bacterial colonization (Miyakawa et al., 2004; Tonetti et al., 2007; Schenkein and Loos, 2013; Fang et al., 2021b; Yuan et al., 2021). Our research directly confirms the association between heart failure and periodontitis, especially moderate/severe periodontitis, and provides clinical evidence for exploring specific mechanisms. The biological mechanism of periodontitis increasing the risk of heart failure presents a promising research field, and it may innovate risk stratification of heart failure and provide ideas for new intervention measures.

Although this study is currently the first to evaluate the incidence of heart failure in patients with periodontitis in the American population, it still has some limitations. First, because of the limitations of data from cross-sectional studies, it is impossible to draw clear conclusions about causality. NHANES III has used the PMPE protocol to examine two quadrants of the oral cavity to assess probing depth and loss of attachment at two fixation sites of teeth (mid- and mesio-buccal) in adult males ≥35 years old. It may lead to underestimation of periodontitis (Eke et al., 2010). Second, there are many confounding factors, such as gender, age, race, and BMI*.* We used standardized research methods and adjusted for different confounders using three models to control important possible confounding factors. Third, hypertension is a common risk factor for periodontitis and heart failure, but we could not obtain detailed hypertension classification for subgroup analysis. Fourth, the incidence of heart failure in patients with periodontitis has only been explored, and the specific types of heart failure have not been evaluated.

In summary, the results of this study show that participants with moderate/severe periodontitis have a higher risk of heart failure. This research supports the literature on the relationship between oral health and cardiovascular disease, and the underlying mechanism should be explored to help inform early interventions for heart failure.

## Data Availability

The original contributions presented in the study are included in the article/Supplementary Material, further inquiries can be directed to the corresponding authors.

## References

[B1] Adamo L. Rocha-Resende C. Prabhu S. D. Mann D. L. (2020). Reappraising the Role of Inflammation in Heart Failure. Nat. Rev. Cardiol. 17 (5), 269–285. 10.1038/s41569-019-0315-x 31969688

[B2] Al-Taweel A. M. Raish M. Perveen S. Fawzy G. A. Ahmad A. Ansari M. A. (2017). Nepeta Deflersiana Attenuates Isoproterenol-Induced Myocardial Injuries in Rats: Possible Involvement of Oxidative Stress, Apoptosis, Inflammation through Nuclear Factor (NF)-κB Downregulation. Phytomedicine 34, 67–75. 10.1016/j.phymed.2017.08.003 28899512

[B3] Albandar J. M. Brunelle J. A. Kingman A. (1999). Destructive Periodontal Disease in Adults 30 Years of Age and Older in the United States, 1988-1994. J. Periodontol. 70 (1), 13–29. 10.1902/jop.1999.70.1.13 10052767

[B4] Arrigo M. Jessup M. Mullens W. Reza N. Shah A. M. Sliwa K. (2020). Acute Heart Failure. Nat. Rev. Dis. Primers 6 (1), 16. 10.1038/s41572-020-0151-7 32139695 PMC7714436

[B5] Bäck M. Hansson G. K. (2015). Anti-inflammatory Therapies for Atherosclerosis. Nat. Rev. Cardiol. 12 (4), 199–211. 10.1038/nrcardio.2015.5 25666404

[B6] Braunwald E. (2013). Heart Failure. JACC: Heart Fail. 1 (1), 1–20. 10.1016/j.jchf.2012.10.002 24621794

[B7] Carrizales-Sepúlveda E. F. Ordaz-Farías A. Vera-Pineda R. Flores-Ramírez R. (2018). Periodontal Disease, Systemic Inflammation and the Risk of Cardiovascular Disease. Heart Lung Circ. 27 (11), 1327–1334. 10.1016/j.hlc.2018.05.102 29903685

[B8] Chen D.-Y. Lin C.-H. Chen Y.-M. Chen H.-H. (2016). Risk of Atrial Fibrillation or Flutter Associated with Periodontitis: A Nationwide, Population-Based, Cohort Study. PLoS One 11 (10), e0165601. 10.1371/journal.pone.0165601 27798703 PMC5087888

[B9] Christiansen M. N. Køber L. Weeke P. Vasan R. S. Jeppesen J. L. Smith J. G. (2017). Age-Specific Trends in Incidence, Mortality, and Comorbidities of Heart Failure in Denmark, 1995 to 2012. Circulation 135 (13), 1214–1223. 10.1161/circulationaha.116.025941 28174193

[B10] Clark K. A. A. Reinhardt S. W. Chouairi F. Miller P. E. Kay B. Fuery M. (2022). Trends in Heart Failure Hospitalizations in the US from 2008 to 2018. J. Card. Fail. 28 (2), 171–180. 10.1016/j.cardfail.2021.08.020 34534665

[B11] Conrad N. Judge A. Tran J. Mohseni H. Hedgecott D. Crespillo A. P. (2018). Temporal Trends and Patterns in Heart Failure Incidence: a Population-Based Study of 4 Million Individuals. The Lancet 391 (10120), 572–580. 10.1016/s0140-6736(17)32520-5 PMC5814791 29174292

[B12] Czesnikiewicz-Guzik M. Osmenda G. Siedlinski M. Nosalski R. Pelka P. Nowakowski D. (2019). Causal Association between Periodontitis and Hypertension: Evidence from Mendelian Randomization and a Randomized Controlled Trial of Non-surgical Periodontal Therapy. Eur. Heart J. 40 (42), 3459–3470. 10.1093/eurheartj/ehz646 31504461 PMC6837161

[B13] Dick S. A. Epelman S. (2016). Chronic Heart Failure and Inflammation. Circ. Res. 119 (1), 159–176. 10.1161/circresaha.116.308030 27340274

[B14] Eke P. I. Thornton-Evans G. O. Wei L. Borgnakke W. S. Dye B. A. (2010). Accuracy of NHANES Periodontal Examination Protocols. J. Dent Res. 89 (11), 1208–1213. 10.1177/0022034510377793 20858782

[B15] Ezzati T. M. Massey J. T. Waksberg J. Chu A. Maurer K. R. (1992). Sample Design: Third National Health and Nutrition Examination Survey. Vital Health Stat. 2 2 (113), 1–35. 1413563

[B16] Fang C. Wu L. Zhao M.-J. Deng T. Gu J.-M. Guo X.-P. (2021a). Periodontitis Exacerbates Benign Prostatic Hyperplasia through Regulation of Oxidative Stress and Inflammation. Oxidative Med. Cell Longevity 2021, 1–11. 10.1155/2021/2094665 PMC8545573 34707774

[B17] Fang C. Wu L. Zhu C. Xie W. Z. Hu H. Zeng X. T. (2021b). A Potential Therapeutic Strategy for Prostatic Disease by Targeting the Oral Microbiome. Med. Res. Rev. 41 (3), 1812–1834. 10.1002/med.21778 33377531 PMC8246803

[B18] Fröhlich H. Herrmann K. Franke J. Karimi A. Täger T. Cebola R. (2016). Periodontitis in Chronic Heart Failure. Tex. Heart Inst. J. 43 (4), 297–304. 10.14503/thij-15-5200 27547136 PMC4979384

[B19] Gao Y. Ren C. Li X. Yu W. Li S. Li H. (2021). Ischemic Conditioning Ameliorated Hypertension and Vascular Remodeling of Spontaneously Hypertensive Rat via Inflammatory Regulation. Aging Dis. 12 (1), 116–131. 10.14336/ad.2020.0320 33532132 PMC7801289

[B20] GBD 2017 Disease and Injury Incidence and Prevalence Collaborators (2018). Global, Regional, and National Incidence, Prevalence, and Years Lived with Disability for 354 Diseases and Injuries for 195 Countries and Territories, 1990-2017: a Systematic Analysis for the Global Burden of Disease Study 2017. Lancet 392 (10159), 1789–1858. 10.1016/s0140-6736(18)32279-7 30496104 PMC6227754

[B21] Groenewegen A. Rutten F. H. Mosterd A. Hoes A. W. (2020). Epidemiology of Heart Failure. Eur. J. Heart Fail. 22 (8), 1342–1356. 10.1002/ejhf.1858 32483830 PMC7540043

[B22] Gu X.-M. Yao S.-B. He Z.-j. Wang Y.-G. Li Z.-H. (2020). Meta-analysis of the success Rate of Heartbeat Recovery in Patients with Prehospital Cardiac Arrest in the Past 40 Years in China. Mil. Med Res 7 (1), 34. 10.1186/s40779-020-00263-7 32631439 PMC7339510

[B23] Guzik T. J. Touyz R. M. (2017). Oxidative Stress, Inflammation, and Vascular Aging in Hypertension. Hypertension 70 (4), 660–667. 10.1161/hypertensionaha.117.07802 28784646

[B24] Huang S. Frangogiannis N. G. (2018). Anti‐inflammatory Therapies in Myocardial Infarction: Failures, Hopes and Challenges. Br. J. Pharmacol. 175 (9), 1377–1400. 10.1111/bph.14155 29394499 PMC5901181

[B25] Leng W.-D. Zeng X.-T. Kwong J. S. W. Hua X.-P. (2015). Periodontal Disease and Risk of Coronary Heart Disease: An Updated Meta-Analysis of Prospective Cohort Studies. Int. J. Cardiol. 201, 469–472. 10.1016/j.ijcard.2015.07.087 26313869

[B26] Luo L. S. Luan H. H. Wu L. Shi Y. J. Wang Y. B. Huang Q. (2021). Secular Trends in Severe Periodontitis Incidence, Prevalence and Disability‐adjusted Life Years in Five Asian Countries: A Comparative Study from 1990 to 2017. J. Clin. Periodontol. 48 (5), 627–637. 10.1111/jcpe.13447 33709419

[B27] Ma L.-L. Qiu Y. Song M.-N. Chen Y. Qu J.-X. Li B.-H. (2019). Clinical Trial Registration and Reporting: Drug Therapy and Prevention of Cardiac-Related Infections. Front. Pharmacol. 10, 757. 10.3389/fphar.2019.00757 31333470 PMC6624234

[B28] Miyakawa H. Honma K. Qi M. Kuramitsu H. K. (2004). Interaction of Porphyromonas Gingivalis with Low-Density Lipoproteins: Implications for a Role for Periodontitis in Atherosclerosis. J. Periodontal Res. 39 (1), 1–9. 10.1111/j.1600-0765.2004.00697.x 14687221

[B29] Moriya J. (2019). Critical Roles of Inflammation in Atherosclerosis. J. Cardiol. 73 (1), 22–27. 10.1016/j.jjcc.2018.05.010 29907363

[B30] Mucci L. A. Hsieh C.-c. Williams P. L. Arora M. Adami H.-O. de Faire U. (2009). Do genetic Factors Explain the Association between Poor Oral Health and Cardiovascular Disease? A Prospective Study Among Swedish Twins. Am. J. Epidemiol. 170 (5), 615–621. 10.1093/aje/kwp177 19648170 PMC2732988

[B31] National Center for Health Statistics (2017). NCHS Research Ethics Review Board (ERB) Approval [Online]. Available at: https://www.cdc.gov/nchs/nhanes/irba98.htm (Accessed 1129, , 2017).

[B32] National Center for Health Statistics (1994). Plan and Operation of the Third National Health and Nutrition Examination Survey, 1988-94. Series 1: Programs and Collection Procedures. Vital Health Stat. 1 1 (32), 1–407. 7975354

[B33] Page R. C. Eke P. I. (2007). Case Definitions for Use in Population-Based Surveillance of Periodontitis. J. Periodontol. 78 (7 Suppl. l), 1387–1399. 10.1902/jop.2007.060264 17608611

[B34] Parikh A. Natarajan S. Lipsitz S. R. Katz S. D. (2011). Iron Deficiency in Community-Dwelling US Adults with Self-Reported Heart Failure in the National Health and Nutrition Examination Survey III. Circ. Heart Fail. 4 (5), 599–606. 10.1161/circheartfailure.111.960906 21705484 PMC3180903

[B35] Pieske B. Tschöpe C. de Boer R. A. Fraser A. G. Anker S. D. Donal E. (2019). How to Diagnose Heart Failure with Preserved Ejection Fraction: the HFA-PEFF Diagnostic Algorithm: a Consensus Recommendation from the Heart Failure Association (HFA) of the European Society of Cardiology (ESC). Eur. Heart J. 40 (40), 3297–3317. 10.1093/eurheartj/ehz641 31504452

[B36] Pink C. Kocher T. Meisel P. Dörr M. Markus M. R. P. Jablonowski L. (2015). Longitudinal Effects of Systemic Inflammation Markers on Periodontitis. J. Clin. Periodontol. 42 (11), 988–997. 10.1111/jcpe.12473 26472626

[B37] Prabhu S. D. Frangogiannis N. G. (2016). The Biological Basis for Cardiac Repair after Myocardial Infarction. Circ. Res. 119 (1), 91–112. 10.1161/circresaha.116.303577 27340270 PMC4922528

[B38] Roth G. A. Forouzanfar M. H. Moran A. E. Barber R. Nguyen G. Feigin V. L. (2015). Demographic and Epidemiologic Drivers of Global Cardiovascular Mortality. N. Engl. J. Med. 372 (14), 1333–1341. 10.1056/NEJMoa1406656 25830423 PMC4482354

[B39] Sanz M. Herrera D. Kebschull M. Chapple I. Jepsen S. Berglundh T. (2020a). Treatment of Stage I-III Periodontitis-The EFP S3 Level Clinical Practice Guideline. J. Clin. Periodontol. 47 (Suppl. 22Suppl 22), 4–60. 10.1111/jcpe.13290 32383274 PMC7891343

[B40] Sanz M. Marco Del Castillo A. Jepsen S. Gonzalez‐Juanatey J. R. D’Aiuto F. Bouchard P. (2020b). Periodontitis and Cardiovascular Diseases: Consensus Report. J. Clin. Periodontol. 47 (3), 268–288. 10.1111/jcpe.13189 32011025 PMC7027895

[B41] Sardu C. Paolisso G. Marfella R. (2020). Inflammatory Related Cardiovascular Diseases: From Molecular Mechanisms to Therapeutic Targets. Cpd 26 (22), 2565–2573. 10.2174/1381612826666200213123029 32053065

[B42] Sattler E. L. P. Ishikawa Y. Trivedi-Kapoor R. Zhang D. Quyyumi A. A. Dunbar S. B. (2019). Association between the Prognostic Nutritional Index and Dietary Intake in Community-Dwelling Older Adults with Heart Failure: Findings from NHANES III. Nutrients 11 (11), 2608. 10.3390/nu11112608 PMC6893765 31683657

[B43] Schenkein H. A. Loos B. G. (2013). Inflammatory Mechanisms Linking Periodontal Diseases to Cardiovascular Diseases. J. Clin. Periodontol. 40 (Suppl. 140 14), S51–S69. 10.1111/jcpe.12060 23627334 PMC4554326

[B44] Schenkein H. A. Papapanou P. N. Genco R. Sanz M. (2020). Mechanisms Underlying the Association between Periodontitis and Atherosclerotic Disease. Periodontol. 2000 83 (1), 90–106. 10.1111/prd.12304 32385879

[B45] Schiattarella G. G. Sequeira V. Ameri P. (2020). Distinctive Patterns of Inflammation across the Heart Failure Syndrome. Heart Fail. Rev. 26, 1333–1344. 10.1007/s10741-020-09949-5 32219614

[B46] Seferović P. M. Vardas P. Jankowska E. A. Maggioni A. P. Timmis A. Milinković I. (2021). The Heart Failure Association Atlas: Heart Failure Epidemiology and Management Statistics 2019. Eur. J. Heart Fail. 23 (6), 906–914. 10.1002/ejhf.2143 33634931

[B47] Sharma P. Dietrich T. Ferro C. J. Cockwell P. Chapple I. L. C. (2016). Association between Periodontitis and Mortality in Stages 3-5 Chronic Kidney Disease: NHANES III and Linked Mortality Study. J. Clin. Periodontol. 43 (2), 104–113. 10.1111/jcpe.12502 26717883 PMC5324563

[B48] Smeets M. Vaes B. Mamouris P. Van Den Akker M. Van Pottelbergh G. Goderis G. (2019). Burden of Heart Failure in Flemish General Practices: a Registry-Based Study in the Intego Database. BMJ Open 9 (1), e022972. 10.1136/bmjopen-2018-022972 PMC6326340 30617099

[B49] Sung C. E. Huang R. Y. Cheng W. C. Kao T. W. Chen W. L. (2019). Association between Periodontitis and Cognitive Impairment: Analysis of National Health and Nutrition Examination Survey (NHANES) III. J. Clin. Periodontol. 46 (8), 790–798. 10.1111/jcpe.13155 31152592

[B50] Tan H. Song Y. n. Chen J. Zhang N. Wang Q. Li Q. (2021). Platelet‐Like Fusogenic Liposome‐Mediated Targeting Delivery of miR‐21 Improves Myocardial Remodeling by Reprogramming Macrophages Post Myocardial Ischemia‐Reperfusion Injury. Adv. Sci. 8 (15), 2100787. 10.1002/advs.202100787 PMC8336489 34137511

[B51] Tonetti M. S. D'Aiuto F. Nibali L. Donald A. Storry C. Parkar M. (2007). Treatment of Periodontitis and Endothelial Function. N. Engl. J. Med. 356 (9), 911–920. 10.1056/NEJMoa063186 17329698

[B52] van der Meer P. Gaggin H. K. Dec G. W. (2019). ACC/AHA versus ESC Guidelines on Heart Failure. J. Am. Coll. Cardiol. 73 (21), 2756–2768. 10.1016/j.jacc.2019.03.478 31146820

[B53] Wood N. Johnson R. B. (2004). The Relationship between Tomato Intake and Congestive Heart Failure Risk in Periodontitis Subjects. J. Clin. Periodontol. 31 (7), 574–580. 10.1111/j.1600-051X.2004.00531.x 15191595

[B54] Yan Z. Qi Z. Yang X. Ji N. Wang Y. Shi Q. (2021). The NLRP3 Inflammasome: Multiple Activation Pathways and its Role in Primary Cells during Ventricular Remodeling. J. Cell Physiol. 236 (8), 5547–5563. 10.1002/jcp.30285 33469931

[B55] Yu Y. Yao R.-Q. Zhang Y.-F. Wang S.-Y. Xi W. Wang J.-N. (2021). Is Oxygen Therapy Beneficial for Normoxemic Patients with Acute Heart Failure? A Propensity Score Matched Study. Mil. Med Res 8 (1), 38. 10.1186/s40779-021-00330-7 34238369 PMC8268364

[B56] Yuan S. Fang C. Leng W.-D. Wu L. Li B.-H. Wang X.-H. (2021). Oral Microbiota in the Oral-Genitourinary axis: Identifying Periodontitis as a Potential Risk of Genitourinary Cancers. Mil. Med Res 8 (1), 54. 10.1186/s40779-021-00344-1 34588004 PMC8480014

[B57] Zeng X.-T. Leng W.-D. Lam Y.-Y. Yan B. P. Wei X.-M. Weng H. (2016). Periodontal Disease and Carotid Atherosclerosis: A Meta-Analysis of 17,330 Participants. Int. J. Cardiol. 203, 1044–1051. 10.1016/j.ijcard.2015.11.092 26638053

[B58] Zhao M.-J. Qiao Y.-X. Wu L. Huang Q. Li B.-H. Zeng X.-T. (2019). Periodontal Disease Is Associated with Increased Risk of Hypertension: A Cross-Sectional Study. Front. Physiol. 10, 440. 10.3389/fphys.2019.00440 31105578 PMC6494953

